# Peer Review of “Mobile App–Reported Use of Traditional Medicine for Maintenance of Health in India During the COVID-19 Pandemic: Cross-sectional Questionnaire Study”

**DOI:** 10.2196/29634

**Published:** 2021-05-07

**Authors:** Samuel Lalmuanawma

**Affiliations:** 1 Department Of Mathematics & Computer Science Mizoram University Mizoram India; 2 Department of Computer Science Government Champhai College Champhai India

**Keywords:** AYUSH Sanjivani app, COVID-19, traditional medicine, Ayurveda, Siddha, Unani, homeopathy

*This is a peer-review report submitted for the paper "Mobile App–Reported Use of Traditional Medicine for Maintenance of Health in India During the COVID-19 Pandemic: Cross-sectional Questionnaire Study"*

## Round 1:

### General Comments

The research was timely and interesting, the claimed results seem acceptable and up to the mark, the work shows originality but slightly weak technical support. However, the authors are advised to consider the following few comments in the current version.

### Specific Comments

#### Major Comments

1. It is believed that the paper reflects on the current COVID-19 (SARS-CoV-2) pandemic; therefore, it is suggested to modify SARS-CoV to SARS-CoV-2 as and when required. However, if the author targeted the 2003 pandemic caused by SARS-CoV, it looks fine, but various web sources posted that the AYUSH Sanjivani app launched recently, in May 2020. Please provide a clear definition for the same.
2. Readers will benefit if the structure of the questionnaire is added in tabular form, indicating the details of the survey including the self-reporting questionnaire. Please add a screenshot of the mobile app if possible.
3. In the current version, the *Data Sources and Data Collection Methods* subsection seems to have redundant information. Please review the second and third paragraphs.
4. It will be beneficial if the structure of the three-layer module used for collecting data mentioned in the *Study Design* section is depicted in detailed tabular form.
5. The current version of the manuscript seems weak, especially in the *Methodology* section. Please review and restructure the structure and content of the same. Considerable information should be added.
6. Please add the tools used for the statistical analysis.
7. The *Results and Discussion* section contain mixed-up information. It is advised to review/restructure the same in the revised version.
8. Throughout the paper, it is unclear where the authors classified/identified a person infected with COVID-19. Please mention clearly how the authors dealt with the same.
9. Technical support seems a little weak. It is advised to provide more support for your findings.
10. The novelty of the manuscript needs improvement.
11. Grammatical errors throughout the manuscript require major proofreading.
12. The references need major revision.

## Round 2:

### General Comments

It is been observed that the revised version of the manuscript addresses every review comment made in Round 1. Therefore, after reviewing, the whole change shows its originality.

However, I am still concerned about the structure of the Questionnaire (which was just added for Round 2)! It does not contain any provable information about patients/subjects with COVID-19. The questionnaire is distributed in a public network, but the self-report does not filter whether the subject is an expert in the medical area. Therefore, the report could be inaccurate. If the questionnaire were selectively collected from medical students or a cohort of patients with COVID-19 taking Ayurvedic medication, there could be some relation with the claimed contributions.

Therefore, I kindly request that the authors change the title and claims to match their results. In the meantime, I do value and enjoy reading your work.

## References

[1] Srikanth N, Rana R, Singhal R, Jameela S, Singh R, Khanduri S, Tripathi A, GoeL S, Chhatre L, Chandra A, Rao BCS, Dhiman KS (2021). Mobile App–Reported Use of Traditional Medicine for Maintenance of Health in India During the COVID-19 Pandemic: Cross-sectional Questionnaire Study. JMIRxMed.

